# Reaching the Unreachable: Effectiveness of Psycho-Education for Improving Mental Health Awareness in Women with Gynecological/Obstetrical Issues in A Low Middle-Income Country

**DOI:** 10.1192/j.eurpsy.2025.2394

**Published:** 2025-08-26

**Authors:** S. Q. Bokhari, A. Manzoor, A. Butt, S. Ijaz, R. Majeed, A. Waheed, W. Batul, A. Saleem

**Affiliations:** 1 Psychiatry, Services Institute of Medical Sciences; 2 Psychology, Services Hospital Lahore, Lahore, Pakistan

## Abstract

**Introduction:**

This research addresses the significant mental health challenges faced by women in Pakistan, where gynecological/obstetrical issues are prevalent, and mental health awareness is often lacking due to cultural stigmatization and misconceptions. Women with conditions such as infertility, and breast and ovarian cancer are at higher risk of psychiatric disorders, and there is a clear correlation between infertility and psychological comorbidity. Cultural beliefs and regional variations further complicate the perception and understanding of mental health in Pakistan.

**Objectives:**

The primary objective of this research is to dispel myths and misconceptions surrounding mental health by providing group psychoeducation on major psychiatric illnesses. The goal is to raise awareness and promote psychiatric and psychological help-seeking behavior among women with gynecological issues.

**Methods:**

This study employs a quantitative approach with a quasi-experimental design. A sample of 55 married female participants from the Gynecology Department of Services Hospital Lahore, underwent pre- and post- psychoeducation assessments. Only married females seeking treatment for gynecological conditions were included, while those already diagnosed or seeking treatment for mental disorders before the onset of gynecological issues were excluded. To assess the participants’ knowledge and beliefs regarding mental illnesses, a self-developed Women Mental Health Checklist was used for pre-and post-assessment. A panel of mental health experts validated the content for the checklist. Psychoeducation material was developed based on established resources, and a panel of experts examined its content validity. A pre-psychoeducation assessment was conducted, followed by psychoeducation sessions that included information about mental disorders associated with gynecological issues. Post-assessment was conducted at a one-month follow-up. SPSS 21 was used to analyze the data.

**Results:**

The repeated measure t-test analysis revealed a statistically significant difference in post-assessment (t (49) = 14.6, p = 0.00) which indicated a strong impact of psychoeducation on post-assessment.

**Conclusions:**

These findings highlight the importance of psychoeducation in promoting help-seeking behavior. However, it is important to understand the study limitations and that future research should explore psychoeducation’s role on a broader level. This research aims to bridge the gap in mental health awareness and help-seeking behavior among women in Pakistan facing gynecological and obstetrical issues, ultimately contributing to improved mental well-being and overall quality of life.

**Disclosure of Interest:**

None Declared

